# 术前短期综合肺康复训练对肺癌合并轻中度慢性阻塞性肺病患者的影响：一项前瞻性随机对照试验

**DOI:** 10.3779/j.issn.1009-3419.2016.11.05

**Published:** 2016-11-20

**Authors:** 玉田 赖, 建华 苏, 梅 杨, 坤 周, 国卫 车

**Affiliations:** 1 610041 成都，四川大学华西医院胸外科 Department of Thoracic Surgery, West China Hospital, Sichuan University, Chengdu 610041, China; 2 610041 成都，四川大学华西医院康复科 Department of Rehabilitation, West China Hospital, Sichuan University, Chengdu 610041, China

**Keywords:** 肺肿瘤, COPD, 肺康复训练, Lung neoplasms, COPD, Pulmonary rehabilitation

## Abstract

**背景与目的:**

围术期肺康复训练计划能够加速肺癌手术患者的术后快速康复，但是其应用方案、时间等仍未统一。肺癌合并慢性阻塞性肺病（chronic obstructive pulmonary disease, COPD）的手术患者，由于其相对较差的肺功能及心肺耐力，一直以来，都是肺部相关并发症的高危人群。本研究旨在探讨术前短期综合肺康复训练对肺癌合并轻中度COPD手术患者的影响。

**方法:**

前瞻性分析2015年3月11日至2015年11月31日四川大学华西医院胸外科行肺叶切除的原发性非小细胞癌合并轻中度COPD患者48例，随机分成实验组和对照组；实验组患者术前完成一周短期综合肺康复方案，包括以雾化吸入普米克令舒、博利康尼和沐舒坦静脉滴注为主的药物康复以及呼吸训练+耐力训练（Nustep）的物理康复；而对照组患者按常规术前准备进行。

**结果:**

最终24例患者纳入实验组，24例患者纳入对照组：实验组患者的术后住院时间[(6.17±2.91) d *vs* (8.08±2.21) d; *P*=0.013]和术后抗生素使用时间[(3.61±2.53) d *vs* (5.36±3.12) d; *P*=0.032]低于对照组，总住院费用[(46, 455.6±5, 080.9) ￥ *vs* (45, 536.0±4, 195.8) ￥, *P*=0.498]、住院材料费用[(21, 155.5±10, 512.1) ￥ *vs* (21, 488.8±3, 470.6) ￥, *P*=0.883]、住院药物费用[(7, 760.3±2, 366.0) ￥ *vs* (6, 993.0±2, 022.5) ￥, *P*=0.223]在两组间均无统计学差异；实验组患者对比训练前后，最大峰值流速（peak expiratory flow, PEF）[(268.40±123.94) L/min *vs* (343.71±123.92) L/min; *P* < 0.001]、6分钟运动距离（6-min walk distance, 6-MWD）[(595.42±106.74) m *vs* (620.90±99.27) m; *P*=0.004]及能量消耗[(59.93±10.61) kcal *vs* (61.03±10.47) kcal; *P*=0.004]提高；术后肺部相关并发症（postoperative pulmonary complications, PPCs）发生率（8.3%, 2/24 *vs* 20.8%, 5/24, *P*=0.416）差异无统计学意义。

**结论:**

术前短期综合肺康复训练能够提高肺癌合并轻中度慢性阻塞性肺病患者心肺耐力，加速患者术后快速康复，可作为术前快速康复计划的重要部分。

## Abstract (en)

**Background and objective:**

Pulmonary rehabilitation (PR) is proposed as an effective strategy to decrease surgical morbidity. However, appropriate rehabilitation plan, initiation time, and optimal duration of PR remain unclear. Lung cancer patients with chronic obstructive pulmonary disease (COPD) are considered high-risk population for postoperative pulmonary complications (PPCs) because of poor lung fitness and cardiopulmonary endurance. This study aims to assess the impact of a one-week, systematic and highly-intensive rehabilitation on surgical lung cancer patients with mild to moderate COPD.

**Methods:**

A randomized controlled trial with 48 subjects was conducted (24 patients each for the intervention and groups). The intervention group received seven days of systematic, integrated and highly-intensive PR before surgical treatment, including: pharmacotherapy with atomizing terbutaline, pulmicort and infusion of ambroxol; and physical rehabilitation with respiratory training and endurance training. The control group underwent standard preoperative care.

**Results:**

For the intervention group, the postoperative length of stay was shorter [(6.17±2.91) d *vs* (8.08±2.21) d; *P*=0.013]; likewise for the duration of antibiotics use [(3.61±2.53) d *vs* (5.36±3.12) d; *P*=0.032]. No significant difference was found between the groups in total in-hospital cost [(46, 455.6±5, 080.9) ￥ *vs* (45, 536.0±4, 195.8) ￥, *P*=0.498], medicine cost [(7, 760.3±2, 366.0) *vs* (6, 993.0±2, 022.5), *P*=0.223], and material cost [(21, 155.5±10, 512.1) ￥ *vs* (21, 488.8±3, 470.6) ￥, *P*=0.883]. In the intervention group, peak expiratory flow [(268.40±123.94) L/min *vs* (343.71±123.92) L/min; *P* < 0.001], 6-min walk distance (6-MWD) [(595.42±106.74) m *vs* (620.90±99.27) m; *P*=0.004], and energy consumption [(59.93±10.61) kcal *vs* (61.03±10.47) kcal; *P*=0.004] were statistically different after the seven-day exercise, compared with those on the first day. Finally, for the intervention group the incidence of PPCs (8.3%, 2/24 *vs* 20.8%, 5/24, 20.8%; *P*=0.416) were lower.

**Conclusion:**

The systematic and highly-intensive pulmonary rehabilitation combining abdominal respiration training, respiratory exercise with incentive spirometry, and aerobic exercise could improve the cardiorespiratory endurance of lung cancer patients with mild to moderate COPD. The proposed program may be a practicable preoperative strategy.

肺癌术后并发症（postoperative pulmonary complications, PPCs）是导致肺癌患者肺叶切除术后住院时间延长、费用增高甚至死亡的主要原因。目前数据表明，肺叶切除术后肺部并发症的发生率在2%-40%之间，平均25%左右^[1-5]^。近年的研究结果^[6-9]^提示我们手术后肺部并发症的发生（尤其是术后肺部感染）与肺癌患者术前就存在高危因素（如长期吸烟、肺功能差等）有关，围术期针对肺癌合并高危因素患者的肺康复治疗，可以有效的降低术后肺部相关并发症。对合并慢性阻塞性肺病（chronic obstructive pulmonary disease, COPD）的肺癌患者进行物理康复（呼吸训练、有氧运动等）可以增加患者运动耐力，减轻呼吸困难症状和疲劳感。甚至在一次康复计划完成后获益还将持续，即使康复计划结束了获益也不会停止，明显改善患者肺功能。针对肺癌合并COPD患者手术前后应用肺康复治疗的研究，不但能够有效降低术后并发症，进而减少住院时间及费用，有望为更多的患者进行手术前后期肺康复训练提供指导。

本研究旨在分析术前短期综合肺康复训练对肺癌合并轻中度慢性阻塞性肺病患者的影响，探讨其在加速患者术后快速康复的应用价值。

## 1 资料与方法

### 1.1 研究对象

前瞻性纳入2015年3月-2015年11月四川大学华西医院胸外科非小细胞肺癌（non-small cell lung cancer, NSCLC）手术患者，按纳入与排除标准筛选、实验知情告知后，最终48例患者被纳入实验。纳入标准：①术后病理诊断为原发性NSCLC；②手术方式为肺切除+系统淋巴结清扫术；③术前无心律失常、心肌梗死等心脏病病史者；④诊断为轻、中度COPD者，且近一月未急性发作，轻、中度COPD的诊断标准依据内《慢性阻塞性肺疾病诊治指南（2013年修订版）》，轻度定义为一秒用力呼气容积（forced expiratory volume in one second, FEV1）/用力肺活量（forced vital capacity, FVC） < 70%且FEV1≥80%预计值，伴或不伴慢性症状；中度为FEV1/FVC < 70%且50%≤FEV1 < 80%预计值，伴或不伴慢性症状。排除标准：①术后诊断为NSCLC除外者；②手术方式为全肺切除者^[10]^；③未按要求完成一周综合肺康复训练者。最终纳入实验组24例，对照组24例。

### 1.2 随机化方法

采用随机数字表法对患者进行随机分组，患者分组由专业统计师实施。实验人员执行肺康复训练方案，观察并记录患者训练情况，数据由统计师搜集、整理、分析。

### 1.3 实验伦理

本实验已通过四川大学华西医院临床试验与生物医学专委会伦理（2015-176）、世界卫生组织国际临床试验注册平台（World Health Organization International Clinical Trial Registration Platform, WHO ICTRP）一级注册中心中国临床实验中心审核，UIN编码为ChiCTR-IOR-16008109。

### 1.4 实验流程

整个过程均在患者入院期间进行。患者入院后，告知肺癌合并轻中度慢性阻塞性肺病患者实验相关问题，签署实验知情同意书。同意参与实验的患者进入随机分组并进行心肺功能评估（评估内容详细见下述）；分组后，实验组患者进行1周术前短期综合肺康复训练（见下述），完成康复训练方案后，患者再次进行心肺功能评估并进入手术流程；对照组患者则遵照常规术前准备进行。

#### 1.4.1 心肺功能评估

整个评估过程在康复训练中心室内（温度：20 ℃-24 ℃）进行，调整功率自行车（konica minolta pulsox-300, endorphin, USA）阻力为27瓦，嘱患者6 min之内尽可能快速的运动，患者因心累、气紧等原因难以耐受时可减慢速度或暂停运动，恢复后继续运动；从患者静息开始检测心律和血氧饱和度到运动结束停止检测，检测项目包括BORG呼吸困难评分，患者6分钟运动距离（6 min moving distance, 6-MMD）及能量消耗，呼气峰值流速（peak expiratory flow, PEF）等。实验组患者锻炼前后须进行心肺功能评估，对比锻炼前后心肺功能情况。

#### 1.4.2 肺康复训练

药物康复治疗内容如下：①雾化吸入支气管扩张剂和糖皮质激素治疗：博利康尼2 mL+普米克令舒4 mL，每天2次，雾化吸入；②祛痰治疗：沐舒坦（盐酸氨溴索）注射液30 mg，每天2次，静滴。物理康复内容如下：（1）呼吸训练：①腹式呼吸训练：患者取平卧位，集中精神，全身放松，经鼻缓慢深吸气到最大肺容量后稍屏气，然后用口缓慢呼气，吸气时膈肌下降，腹部外凸；呼气时膈肌上升，腹部内凹。连续进行20次-30次（总时间约15 min-30 min），早晚一次。②吸气训练器训练（VOLDYNE5000呼吸训练器）：患者取坐位，正常呼气后用嘴含紧吸气嘴，以大的吸气量把小球吸上筒腔的顶端不动，屏气2 s-3 s，然后移开吸气嘴，缩唇慢呼气，重复练习，每2 h一组，每组12次-20次^[8]^。（2）下肢耐力训练：①NUSTEP锻炼：患者自行调控速度，在承受范围内逐步加快运动速度及NUSTEP功率。运动量控制在BORG评分5分-7分之间，若在运动过程中有明显气促、腿疲倦、血氧饱和度下降（< 88%）或其他并存疾病引起身体不适，告诉患者休息，待恢复原状后再继续进行训练。每次约15 min-20 min，1次/天，疗程为1周。②爬楼梯训练：在专业治疗师陪同下进行，在运动过程中调整呼吸节奏，采用缩唇呼吸，用力时呼气，避免闭气，稍感气短时可坚持进行，若有明显呼吸困难，可做短暂休息，尽快继续运动。每次约15 min-30 min，1次/天，疗程为1周^[8]^。

#### 1.4.3 欧洲癌症研究与治疗组织癌症治疗功能性量表（European Organization of Researchment and Treatment of Cancer-Quality of Life Questionnaire-Lung Cancer, EORTC-QLQ-LC43）评分

该量表包括C-30与其肺癌子模块LC13（lung cancer 13）共同组成，包括社会、躯体、情感、角色和认知5个维度。患者总体健康水平、躯体功能、情感功能使用EORTC-QLQ-C30量表评估，分值标准化后，分值越高，患者的该项功能越好；患者呼吸困难程度EORTC-QLQ-LC13量表评估，分值标准化后，分值越高，表示呼吸困难程度越严重^[11]^。

### 1.5 干预方法

符合上述标准的入组患者，随机采用下述2种干预方案：实验组：术前行肺康复治疗，包括物理康复+药物康复。对照组术前不行上述方案的肺康复治疗。若患者出现相应的症状和体征，则根据COPD指南，按照常规模式进行干预[如：β2受体激动剂（如沙丁胺醇）、氨茶碱等药物治疗或（和）氧疗等]。

### 1.6 观察指标

#### 1.6.1 主要终点指标--肺部并发症发生率

PPCs定义为：（1）肺炎，肺炎的诊断必须至少满足以下中的3条标准：①胸部平片提示肺部渗出、实变影；②发热38 ℃以上；③白细胞（white blood cell, WBC） > 10, 000/mm^3^或 < 3, 000/mm^3^；④痰液中查见病原菌或支气管镜查见脓性分泌物；（2）痰液淤积，定义为肺叶或全肺肺不张，需要纤支镜吸痰；（3）术后胸腔积气：胸部X线片提示胸腔积气 > 30%；（4）术后胸腔积液：胸部X线片提示胸腔积液中量以上；（5）急性呼吸衰竭，定义为术后持续通气支持 > 12 h或需要重新插管；（6）慢性呼吸衰竭，定义为出院后持续氧气吸入时间长于1个月。

#### 1.6.2 次要终点指标

包括锻炼前后PEF值、6-MMD及能量消耗、锻炼前后血气分析值、EORTC-LC43值；患者住院期间住院费用、术后住院天数、抗生素使用时间等。

### 1.7 统计学方法

应用SPSS 19.0软件分析结果，计数资料用实际例数及百分比表示，采用独立样本的卡方检验或*FISH*检验，计量资料采用均数±标准差（Mean±SD）表示，采用*t*检验，以*P* < 0.05为差异有统计学意义。

## 2 结果

### 2.1 基本资料

最终48例患者被纳入实验[年龄：（63.43±7.64）岁]，其中24例为实验组，24例为对照组；58.3%（28/48）为男性，41.7%（20/48）为女性；频繁吸烟患者14例（29.2%, 14/48），非吸烟者24例（50.0%, 24/48），曾经吸烟现戒烟1年以上者10例（20.8%, 10/48）；轻度COPD患者14例（29.2%），中度COPD患者34例（70.8%），组间差异无统计学意义（*P*=0.525）（表 1）。

**1:** 患者基线资料 Baseline of the patients

	Intervention group	Control group	*P* value
Age	63.13±6.26	64.04±8.94	0.683
Gender			0.763
Female	9 (37.5%)	11 (45.8%)	
Male	15 (62.5%)	13 (54.2%)	
Lung function			
FVC (L)	2.90±0.87	2.74±0.77	0.507
FEV1	2.42±0.79	2.51±0.74	0.443
MVV	93.42±27.49	87.69±26.79	0.220
Dlco SB	22.62±4.28	21.83±4.35	0.881
COPD classification			0.525
Mild	6 (25.0%)	8 (33.3%)	
Moderate	18 (75.0%)	16 (66.7%)	
Smoking status			0.461
Current-smoker	7 (29.2%)	7 (29.2%)	
Non-smoker	11 (45.8%)	13 (54.2%)	
Ever-smoker	6 (25.0%)	4 (16.6%)	
Pathological stage			0.366
Stage 0 or Ⅰ	8 (33.3%)	13 (54.2%)	
Stage Ⅱ	13 (54.2%)	8 (33.3%)	
Stage Ⅲ	2 (8.3%)	3 (12.5%)	
Stage Ⅳ	1 (4.2%)	0 (0)	
Tumor pathological type			0.955
Adenocarcinoma	11 (45.8%)	10 (41.7%)	
Squamous carcinoma	11 (45.8%)	12 (50.0%)	
Adenosquamous carcinoma	2 (8.3%)	2 (8.3%)	
Tumor grade			0.318
Poorly differentiated	13 (54.1%)	10 (41.7%)	
Between moderate and poor	6 (25.0%)	6 (25.0%)	
Moderate differentiated	4 (16.7%)	2 (8.3%)	
Between moderate and high	1 (4.2%)	5 (20.8%)	
High-differentiated	0 (0)	1 (4.2%)	
COPD: chronic obstructive pulmonary disease; FEV1: forced expiratory volume in one second; FVC: forced vital capacity; MVV: maximum ventilatory volume; Dlco SB: diffusion capacity for carbon monoxide of the lung (single breath).			

### 2.2 住院时间及费用

住院时间方面，实验组患者的术后住院时间低于对照组[(6.17±2.91) d *vs* (8.08±2.21) d, *P*=0.013]，而术前住院时间[(8.25±1.39) d *vs* (7.67±3.37) d, *P*=0.439]和总住院时间[(14.04±3.20) d *vs* (15.75±3.22) d, *P*=0.072]无明显差异；抗生素使用时间方面，实验组患者术后抗生素使用时间低于对照组[(3.61±2.53) d *vs* (5.36±3.12) d, *P*=0.032]；住院费用方面，总住院费用[(46, 455.6±5, 080.9) ¥ *vs* (45, 536.0±4, 195.8) ¥, *P*=0.498]、住院材料费用[(21, 155.5±10, 512.1) ¥ *vs* (21, 488.8±3, 470.6) ¥, *P*=0.883]、住院药物费用[(7760.3±2366.0) ¥ *vs* (6, 993.0±2, 022.5) ¥, *P*=0.223]组间均无统计学差异（表 2）。

**2:** 患者临床特征 Clinical characteristics

	Intervention group	Control group	*P* value
Surgical approach			0.540
VATS	17 (70.8%)	15 (62.5%)	
Open	7 (29.2%)	9 (37.5%)	
Resection type			0.366
Segmental or wedge	12 (50.0%)	8 (33.3%)	
Lobectomy	8 (33.3%)	13 (54.2%)	
Combined lobectomy	3 (12.5%)	2 (8.3%)	
Other	1 (4.2%)	1 (4.2%)	
Length of stay			
Total (d)	14.04±3.20	15.75±3.22	0.072
Preoperative (d)	8.25±1.39	7.67±3.37	0.439
Postoperative (d)	6.17±2.91	8.08±2.21	0.013
In-hospital expense			
Total cost (¥)	46, 455.6±5, 080.9	45, 536.0±4, 195.8	0.498
Drug cost (¥)	7, 760.3±2, 366.0	6, 993.0±2, 022.5	0.223
Material (¥)	21, 155.5±10, 512.1	21, 488.8±3, 470.6	0.883
Duration of antibiotic use (d)	3.61±2.53	5.36±3.12	0.032
PEF: peak expiratory flow; 6-MMD: 6-min moving distance; VATS: video-assisted thoracic surgery.			

### 2.3 心肺功能评估

实验组患者自身前后对比发现，PEF[(268.40±123.94) L/min *vs* (343.71±123.92) L/min; *P* < 0.001]、6-MMD[(595.42±106.74) m *vs* (620.90±99.27) m; *P*=0.004]及能量消耗[(59.93±10.61) kcal *vs* (61.03±10.47) kcal; *P*=0.004]显著提高；而在运动中BROG呼吸困难指数评分、疲劳指数评分上，差异无统计学意义[(1.52±1.02) *vs* (1.40±0.68), *P*=0.529; (1.04±0.61) *vs* (1.15±0.63), *P*=0.204]（表 3）。

**3:** 实验组康复训练前后的比较 Comparative analysis before and after intervention

	Before the exercise	After the exercise	*P* value
PFE	268.40±123.94	343.71±123.92	< 0.001
6-MMD (m)	595.42±106.74	620.9±99.27	0.004
Index of fatigue in exercise	1.52±1.02	1.40±0.68	0.529
Index of dyspnea in exercise	1.04±0.61	1.15±0.63	0.204
Energy consumption (Kcal)	59.93±10.61	61.03±10.47	0.004
Blood gas analysis			
PCO_2_ (mmHg)	43.06±3.95	41.65±4.20	0.207
PO_2_ (mmHg)	79.51±28.73	75.91±8.77	0.577
SaO_2_ (%)	94.17±6.06	94.82±3.40	0.687
PH	7.39±0.03	7.31±0.03	0.686
QoL evaluation			
Global QoL^*^	67.71±11.33	68.34±12.67	0.912
Physical function^*^	81.13±5.42	80.45±4.74	0.769
Emotional function^*^	83.55±7.53	84.26±7.42	0.211
Dyspnea score†	22.21± 13.91	17.34±11.03	0.067
^*^Higher scores indicate better functioning (scaled from 0-100); †Lower scores indicate less dyspnea (scaled from 0-100). PEF: peak expiratory flow; PO_2_: partial pressure of oxygen; PCO_2_: partial pressure of carbon dioxide; SaO_2_: arterial oxygen saturation; QoL: quality of life.			

### 2.4 训练前后血气分析的比较

PH值[(7.39±0.03) *vs* (7.31±0.03), *P*=0.686]、氧分压（partial pressure of oxygen, PO_2_）值[(79.51±28.73) mmHg *vs* (75.91±8.77) mmHg; *P*=0.577]、二氧化碳分压（partial pressure of carbon dioxide, PCO_2_）值[(43.06±3.95) *vs* (41.65±4.20) mmHg; *P*=0.207]以及动脉血氧饱和度（arterial oxygen saturation, SaO_2_）值[(94.17±6.06) *vs* (94.82±3.40), *P*=0.687]，差异无统计学意义。

### 2.5 训练前后生活质量评价

EORTC-LC43问卷方面，患者总体健康水平[(67.71±11.33) *vs* (68.34±12.67); *P*=0.912]、躯体功能[(81.13±5.42) *vs* (80.45±4.74); *P*=0.769]、情感功能[(83.55±7.53) *vs* (84.26±7.42); *P*=0.211]以及呼吸困难程度[(22.21± 13.91) *vs* (17.34±11.03); *P*=0.067]方面差异均无统计学意义（表 3）。

### 2.6 肺部相关并发症

发生肺部相关并发症的7例（14.6%, 7/48）患者中，发生肺部感染的比例最高（8.3%, 4/48），其他并发症包括持续性肺漏气（6.25%, 3/48），肺不张（4.2%, 2/48）、肺栓塞（2.1%, 1/48）、呼吸衰竭（2.1%, 1/48）。实验组患者肺部相关并发症发生率低于对照组，而差异无统计学意义（8.3%, 2/24 *vs* 20.8%, 5/24; *P*=0.416）（表 4）。

**4:** 两组间PPCs发生率的比较 Comparison of PPCs between groups

	Intervention group	Control group	*P* value
Total	2 (8.3%)	5 (20.8%)	0.416
Pneumonia	2 (8.3%)	2 (8.3%)	
Atelectasis	1 (4.2%)	1 (4.2%)	
Pulmonary embolism	0 (0)	1 (4.2%)	
Respiratory/heart failure or ADRS	0 (0)	1 (4.2%)	
Air leak	1 (4.2%)	2 (8.3%)	
PPCs: postoperative pulmonary complications; ADRS: acute respiratory distress syndrome.			

## 3 讨论

医学技术的进步和医学管理模式的更新使得快速康复外科（fast-track surgery, FTS）或加速康复外科（enhanced recovery after surgery, ERAS）这一理念逐渐被接受并认可。围绕微创技术的围手术期流程优化和多学科协作，成为目前外科领域的一大热点^[12-14]^。近年来，研究^[15]^表明，肺康复在胸外科领域，尤其对加速肺癌手术患者快速康复上，有着较为明显的作用。然而，由于现今肺康复训练方案尚未统一，没有相对一致的方案标准，导致其在应用上存在相当大的差异^[15]^。肺癌合并COPD的手术患者，无论在手术风险上还是在术后康复上，都存在较大的难度，并由于其相对较差的肺功能及心肺耐力，一直以来，都是肺部相关并发症的高危人群^[16, 17]^。

短期综合肺康复训练由药物康复和包括呼吸训练和耐力训练的物理康复组成，呼吸训练使患者更好的掌握腹式呼吸，并通过锻炼呼吸肌促进患者有效咳嗽咳痰，NUSTEP训练增强手术患者心肺耐力，术前药物治疗有效控制、缓解患者COPD病情，均能够为预防或减少肺癌术后肺部相关并发症的发生起到有效帮助，最终达到术后快速康复的目的；尽管在患者术后并发症、住院费用方面组间并无统计学差异，但是实验组患者术后住院时间相比对照组明显缩短，同时抗生素的使用时间也更少，这都提示综合康复在术后快速康复上具有一定的作用和价值。

PEF作为常用肺功能指标之一，较多用于诊断、监测哮喘等呼吸系统疾病。近年多项实验证明其与手术并发症、死亡率的关系，且有研究^[18-20]^表明PEF一定程度上能够反映咳嗽咳痰的能力，可作为一项预测手术预后的有效指标。通过呼吸训练，能够有效锻炼呼吸肌功能，提高患者咳嗽咳痰能力，在患者接受手术打击后，有效促进患者排痰，从而降低术后肺部相关并发症（如肺部感染、肺不张等）发生率^[21]^。经过1周综合高强度肺康复训练后，实验组患者PEF较前有较大提升，差异具有统计学意义，证明其对PEF的提升效果。

心肺评估方面，经过1周综合高强度肺康复训练后，实验组患者6-MMD及能量消耗得到了提高，且与锻炼前相比差异具有统计学意义。这说明术前的康复训练对此类患者的心肺耐力有较大的帮助，而心肺耐力的提高使患者更好的耐受手术打击，手术后也更利于心肺功能的恢复，最终降低术后肺部相关并发症的发生风险，加速患者术后快速康复。实验结果表明实验组患者的肺部相关并发症发生率有低于对照组的趋势，尽管受限于实验样本量较小等原因，二者差异无统计学意义，这一趋势亦从一定程度上反映出综合康复对降低肺部相关并发症发生风险的积极作用。此外，实验结果显示实验组患者短期锻炼前后，血气分析结果、生活质量等方面，无明显的差异，这与相对较短的实验干预时间密切相关，这也提示我们短期的综合康复在这些方面影响的有限性。

本研究还存在一定的不足。由于临床实验的特殊性，实验样本量较小，导致实验结果在相关实验干扰因素（如患者的个体差异）的影响下偏倚较大，同时使得一些实验结果（如术后肺部相关并发症）并不能在统计学上出现意义；此外，对照组患者按常规术前准备，不能排除患者自行进行锻炼（如自己爬楼梯，自己进行呼吸训练，有COPD症状时使用雾化药物治疗等），这些都对实验结果造成干扰；最后，本研究为单中心随机对照研究，其实验结果的可推广性仍需多中心实验进行进一步论证。

术前短期综合肺康复训练能够提高肺癌合并轻中度慢性阻塞性肺病患者心肺耐力，加速患者术后快速康复，可作为术前快速康复计划的重要部分。
